# GLP-1RA and the possible skin aging

**DOI:** 10.1007/s12020-025-04293-w

**Published:** 2025-06-11

**Authors:** Ioanna A. Paschou, Evangelia Sali, Stavroula A. Paschou, Konstantinos I. Tsamis, Melpomeni Peppa, Theodora Psaltopoulou, Electra Nicolaidou, Alexander J. Stratigos

**Affiliations:** 1 1st Department of Dermatology and Venereology, “Andreas Sygros” Hospital, School of Medicine, National and Kapodistrian University of Athens, Athens, Greece; 2 Endocrine Unit and Diabetes Centre, Department of Clinical Therapeutics, Alexandra Hospital, School of Medicine, National and Kapodistrian University of Athens, Athens, Greece; 3 Department of Physiology, Faculty of Medicine, School of Health Sciences, University of Ioannina, Ioannina, Greece; 4 Endocrine Unit and Diabetes Center, Second Department of Internal Medicine, School of Medicine, Attikon University Hospital, National and Kapodistrian University of Athens, Athens, Greece

**Keywords:** Skin, Aging, GLP-1RA, AGEs, Type 2 diabetes, Obesity

## Abstract

“Ozempic face” and facial aging have been observed as side effects in many patients after glucagon like peptide 1 receptor agonists (GLP-1RA) therapy for type 2 diabetes mellitus (T2DM) and obesity. However, those medications can reduce systemic inflammation and possibly promote skin health. The rapid weight loss observed with GLP-1RA has been implicated in facial aging. However, recent evidence suggests further pathophysiological mechanisms for this side effect. The aim of this article is to review the literature and present available data on the possible mechanisms of GLP-1RA on skin aging. Indeed, GLP-1RA may affect other types of skin cells, which may accelerate the process of skin aging itself. More specifically, GLP-1RA can act on adipose-derived stem cells (ADSC) and fibroblasts, that present GLP-1R on their surface. Stimulation of the receptor reduces the ability of ADSC to produce protective cytokines. The absence of those cytokines promotes the production of reactive oxygen species (ROS) and causes oxidative damage on fibroblasts. GLP-1RA also reduce the glucose intake of the ADSC, leading to reduced production of ATP and apoptosis. Finally, the stimulation of GLP-1R on ADSCs reduces indirectly the production of estrogens from dermal white adipose tissues (DWAT), which reduces stimulation of fibroblasts to produce collagen. GLP-1RA can also affect the process of skin aging through interaction with advanced glycation end products (AGEs) and RAGE (receptors of AGEs) activation. In conclusion, many patients receiving GLP-1RA suffer from “Ozempic face” and facial aging. It seems that this complication is not exclusively related to decreased facial fat, but there are more aging mechanisms that have to be elucidated.

## Introduction

Glucagon like peptide 1 receptor agonists (GLP-1RA) are medications used as a treatment for type 2 diabetes mellitus (T2DM) and obesity. GLP-1 and GLP-1RA can regulate glucose levels, by stimulating insulin secretion and reducing the release of glucagon. They can also lead to weight loss by reducing the appetite and delaying gastric emptying [1]. GLP-1RA have been found to be valuable medications for inflammatory skin diseases as well, such as psoriasis, due to their immunological effects [2–4]. However, with the increased popularity of GLP-1RA, the so-called “Ozempic Face” has emerged. “Ozempic face” was coined by a dermatologist, Paul Jarrold Frank, to describe the facial aging that many of his patients who receive GLP-1RA suffer from. This side effect was more noticeable in patients who received the GLP-1RA treatment off-label, as a weight-loss medication. This phenomenon was initially explained by the loss of facial fat, which can lead to excess skin and profound wrinkles. Fat loss in certain areas can alter the proportions and shape of the face, the size of the lips, cheeks and chin, altering the characteristics and thus, resulting in facial aging [5–7]. However, the mechanism behind “Ozempic face” has not been fully understood.

Skin aging is the process in which the quality of dermis and epidermis deteriorates, and the skin appears thinner and drier with profound wrinkles and with less elasticity. Skin aging is characterized by decreased mitotic activity, disfunction of the skin barrier and reduced production of collagen and elastin fibers. The skin cells have decreased proliferative ability, as they cannot re-enter the mitotic circle and eventually, they begin the process of apoptosis [8, 9]. Oxidative stress can also affect skin health due to the deterioration of the anti-oxidative mechanisms and the increased production of reactive oxygen species (ROS), causing DNA damage and oxidation of the cellular membranes. The impairment of the membrane lipids reduces the efficacy of signaling through the membrane. Oxidative stress can also activate the MAP kinase pathway which results in reduced production of procollagen. Finally, oxidative stress can activate NF-kB, which stimulates the production of proinflammatory cytokines and is also involved in collagen metabolism [9].

## Possible mechanisms of skin aging mediated by GLP-1RA

One possible mechanism leading to “Ozempic face” is the rapid loss of fat, which can alter the proportions of the face, resulting in facial aging. “Ozempic face” is not a side effect exclusively linked with semaglutide or other GLP-1RA, but an outcome of rapid weight loss and malnutrition. This is evident in patients who had significant weight loss after bariatric surgery. The histological analysis of the biopsies of those patients revealed structural alteration in the dermis, as weight loss possibly affects the density of collagen and elastic fibers [7, 10–12]. Patients receiving GLP-1RA may also experience deterioration of their skin health and acceleration of skin aging. It is still not clear whether all patients receiving GLP-1RA are bound to suffer from “Ozempic face”, and if this complication is only related to lower fat volume or if there are more mechanisms responsible for this phenomenon.

There is some evidence suggesting that GLP-1RA may accelerate skin aging by targeting cells in the adipose tissue layer and inhibiting their proliferation and metabolic activity. As reported by Ridha et al. [13], GLP-1RA affects the dermal white adipose tissue (DWAT) and the adipose-derived stem cells (ADSCs), and possibly, the hormonal regulation and the muscles of the face. Researchers have found that the GLP-1 receptor is expressed on the DWAT [14]. DWAT is a layer of adipocytes with metabolic activity, immune cells, fibroblasts and ADSCs in the dermis. The DWATs volume has been found to affect the process of skin aging. Aged skin is characterized by the decreased volume of DWAT and therefore, a decreased amount of cells producing collagen. Apart from the decreased production of collagen, there is an increased activity of metalloproteinase-1, an enzyme responsible for collagen disintegration [13].

**Fig. 1:**
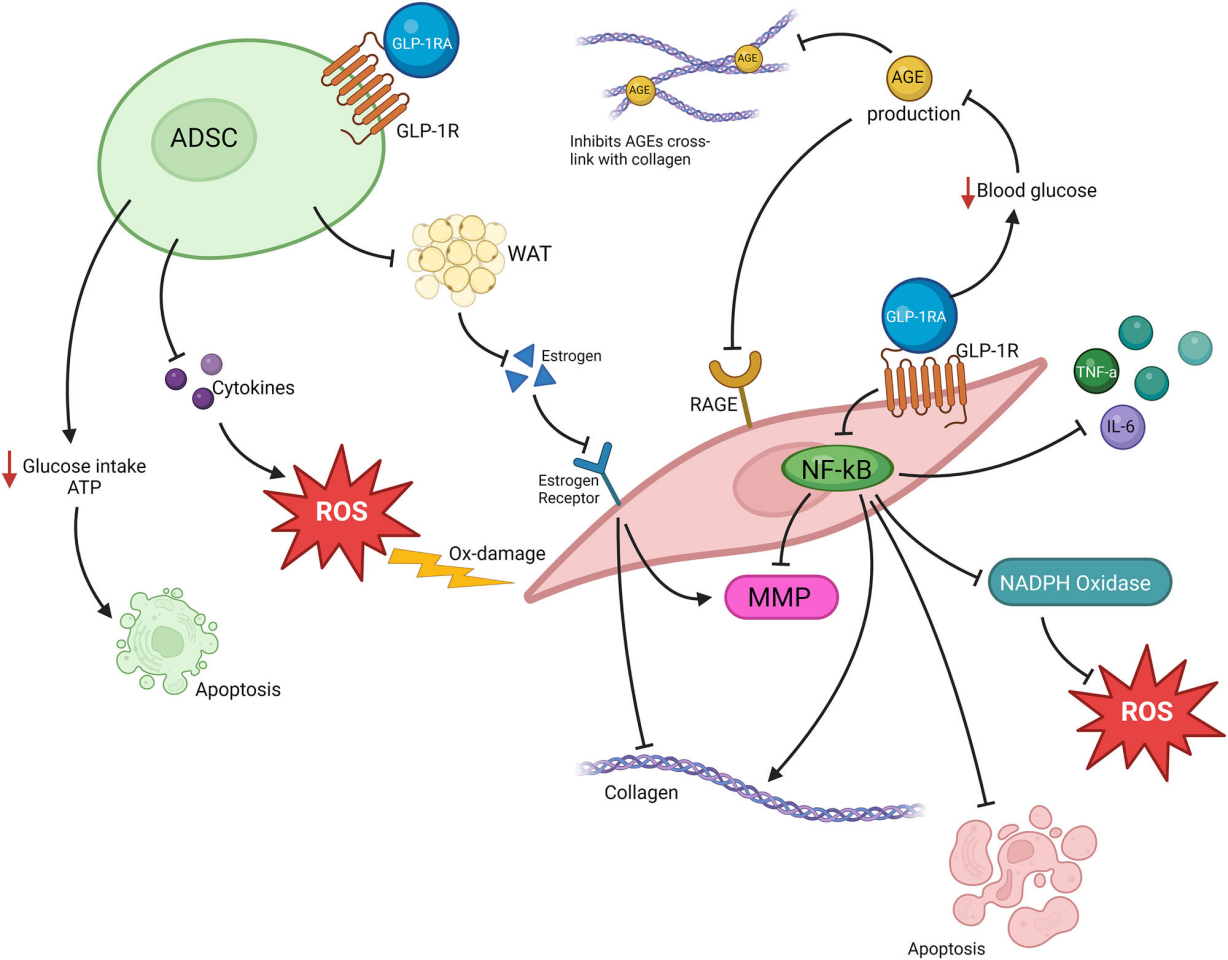
Suggested mechanisms of GLP-1RA on skin aging. GLP-1RA can act on ADSCs and fibroblasts through GLP-1R on their surface. Stimulation of the receptor reduces the ability of the ADSC to produce protective cytokines. The absence of those cytokines promotes the production of ROS and causes oxidative damage on fibroblasts. GLP-1RA also reduces the glucose intake of the ADSC, leading to reduced production of ATP and apoptosis. Finally, the stimulation of GLP-1R on ADSCs, indirectly reduces the production of estrogen from DWAT. The decreased amount of estrogen cannot stimulate fibroblasts to produce collagen. GLP-1RA can also affect the process of skin aging through AGEs. GLP-1RA can reduce the production of AGEs and thus, the cross-link with collagen. Moreover, GLP-1RA can interfere with the stimulating pathway after RAGE activation, by inhibiting NF-kB. That leads to decreased production of inflammatory cytokines, inhibition of NADPH oxidase and ROS production, inhibition of fibroblast apoptosis, production of collagen and decreased activity of MMPs. This figure suggests that there are two conflicted mechanisms in which GLP-1RA can affect skin aging. The effect of GLP-1RA on ADSCs, indicates the deterioration of skin health and the acceleration of skin aging. On the other hand, GLP-1RA, by reducing AGEs production and by inhibiting RAGEs activation, benefits skin health and delays skin aging

ADSCs are mesenchymal cells in the adipose tissue with the ability to differentiate and regenerate. Upon stimulation, ADSCs can rapidly produce cytokines, hormones and growth factors, leading to skin rejuvenation [13]. A study by Kim et al. [15], has indicated that ADSCs have also anti-oxidative effects on fibroblasts. Protection against ROS is probably due to the protective cytokines and other products by ADSCs, such as IGF, which can prevent oxidation damage on fibroblasts. ADSCs also interact with fibroblasts, stimulating the migration of fibroblasts, triggering collagen production and promoting wound healing and skin rejuvenation. The effect of GLP-1RA in skin aging, besides weight loss, is possibly due to GLP-1 receptors on ADSCs. GLP-1RA can activate the receptor located on the surface of the human ADSCs and inhibit proliferation and differentiation of ADSCs [16]. The decreased proliferative ability of the ADSCs can affect the response to oxidative damage, the production of cytokines and the activity of fibroblasts. More specifically, there is an increase in the oxidation of the ADSCs after the stimulation of the receptor [13, 16]. The GLP-1RA can also decrease the migration of fibroblasts and their synthetic ability [13] (Fig. 1).

The GLP-1RA was found to act differently on ADSCs compared to other cells regarding glucose intake. GLP-1 and analogs increase glucose consumption in mature cells whereas they reduce it in progenitor cells and ADSCs, which can lead to decreased production of ATP and energy deficiency. Reduced glucose levels can also increase the production of ROS causing oxidation damage and interfere with signaling pathways and finally lead to apoptosis and necrosis [16] (Fig. 1).

Another effect of the GLP-1RA on the ADSCs is the change of hormones production. The decreased proliferation and activity of the ADSCs reduces the production of estrogen and various growth factors [13]. Studies have indicated that low estrogen levels in the skin can accelerate skin aging [17]. Fibroblasts have estrogen receptors on their surface and when being stimulated, they can increase collagen production [18]. The decreased production of estrogen in patients receiving GLP-1RA inhibits the collagen synthesis and increases the activity of metalloproteinase-1, leading to matrix collagen and elastin degradation. Also, DWAT is a source of estrogen production and treatment with GLP-1RA reduces those aging protective hormones due to DWAT loss [13] (Fig. 1).

Finally, there is some conflicting evidence about the effect of GLP-1RA therapy and muscle loss. A study by Ida et al. [19] has shown that patients receiving GLP-1RA for T2DM have had fat and muscle loss compared to patients receiving placebo. However, other studies have shown a protective role of the GLP-1RA on muscle cells, preventing muscle atrophy and stimulating myogenesis [20, 21].

## Indications that GLP-1RA may promote skin health

Studies on the direct effects of GLP-1RA on skin aging are limited. Although more research is needed, there are some indications that GLP-1RA may delay skin aging and benefit skin health. More specifically, GLP-1RA has been found to reduce chronic inflammation and advanced glycation end products (AGEs) levels, which can protect against cardiovascular disease, aging and age-related diseases [22–24]. AGEs are a result of the glycation reaction between glucose and proteins, nucleic acids and lipids. Chronic hyperglycemia in diabetes increases the production of AGEs, leading to diabetes related complications. Measurement of AGEs in the skin is a non-invasive method of estimating the cardiovascular mortality, the risk for diabetes complications and also the skin health and the aging process [25]. AGEs receptors (RAGE) are located in many tissues including skin. When they are being stimulated, they activate NF-kB which increases the production of pro-inflammatory cytokines such as IL-6 and TNF-a. AGE-RAGE bind also activates NADPH oxidase and increases ROS production [26]. The accumulation of AGEs in the epidermis can impair the function of the skin barrier and obstruct wound healing by increasing the production of matrix metalloproteinase MMP-9. AGEs also affect keratinocytes and melanocytes, leading to the thinning of the stratum corneum and to the increased production of melanin. In the dermis, AGEs can promote fibroblast apoptosis and destruction of fiber contracture. On human fibroblasts, after AGEs bind to the receptors, they increase the production of ROS and NF-kB [25]. AGEs are also able to cross-link with collagen and elastin fibers resulting in the stiffening of the tissue [26]. Further, the accumulation of AGEs in the extracellular matrix increases the activity of metalloproteinases and therefore reduces collagen and elastin synthesis [25].

GLP-1RA as a treatment for T2DM has been reported to reduce AGEs production and thus decrease the risk of diabetes complications. High levels of AGEs have been found to accelerate not only the process of skin aging, but also the vascular aging and the development of neurodegenerative diseases [27, 28]. Treatment with GLP-1RA has been shown to reduce AGEs in the extracellular matrix in the myocardium [28]. GLP-1RA, as a glucose reducing medication, can reduce the glycation process and the production of AGEs. However, GLP-1RA has both anti-inflammatory and antioxidant effects, which can interfere with the signaling pathways of AGEs [29]. In a study by Di et al. [30], it was found that liraglutide can inhibit AGE-induced NF-kB signaling pathway in the vascular smooth muscle cells. In another study by Zhang et al. about osteoarthritis [31], it was found that GLP-1RA can affect AGE-induced inflammation in chondrocytes. Liraglutide has been found to reduce RAGE levels and hinder the production of inflammatory cytokines such as IL-1β, IL-6, IL-12 and TNF-a. Additionally, GLP-1RA reduced the production of various of AGEs-induced catabolic factors (MMP1, MMP3, MMP13, ADAMTS4 and ADAMTS5), preventing matrix degeneration, while promoting anabolism. Last but not least, GLP-1RA reduced apoptosis in chondrocytes. In various studies, there has been found that GLP-1RA can also protect against oxidative damage by reducing the AGEs-induced ROS production [22, 27, 32]. In a randomized controlled trial by Xie et al. [33], dulaglutide managed to significantly reduce AGEs levels in the peripheral blood, and consequently inhibit ROS production, oxidative damage and production of proinflammatory mediators, such as IL-6, TNF-a and CRP (Fig. 1). In the same study, it was found that GLP-1RA can affect progenitor endothelial cells (EPCs). Dulaglutide was proven to be able to promote proliferation and migration of EPCs, as well as inhibit apoptosis.

Contrary to the image of “Ozempic face” due to the possible direct effects of the GLP-1RA on ADSCs and fibroblasts, the reduction of AGES indicates that those medications may benefit skin health. Those two conflicting theories are illustrated in Fig. 1, where GLP-1RA acts on ADSCs resulting in apoptosis, oxidative damage on fibroblasts and decreased collagen production. The figure also displays the reduction of AGES levels, the inhibition of AGES-collagen cross-link and the inhibition of NF-kB and thus the effects on cytokine and collagen production, oxidative stress and cell survival.

Finally, additional studies have indicated that GLP-1RA also affects the endothelial function of the small blood vessels in the skin and subcutaneous tissue. Those studies revealed that GLP-1RA can act directly and indirectly in the arterioles and capillaries and increase microvascular perfusion [34–36]. There are not any published papers linking the increased skin perfusion by GLP-1RA treatment with the skin aging prosses. However, this can lead to increased blood flow, better oxygen and nutrient delivery in the tissues. This may benefit skin health, promote wound healing and skin regeneration, and delay the aging process [37].

## Conclusions

In conclusion, GLP-1RA has been found to delay the aging process by reducing the systemic inflammation and the AGEs levels in the body. That indicates a possible improvement in skin health, as well. Yet, some patients who receive those medications suffer from facial aging. It remains to be elucidated whether the process of aging is accelerating only on the skin while GLP-1RA is delaying the systemic aging, and if so, which exact pathophysiological mechanisms are hidden behind this complication.

## Data Availability

No datasets were generated or analysed during the current study.
